# Supplementary material for: Global Spatio-temporal Patterns of Influenza in the Post-pandemic Era
Source: Sci Rep. 2015 Jun 5;5:11013. doi: 10.1038/srep11013 (PMC4457022; doi:10.1038/srep11013)
Supplement: Supplementary Information [file srep11013-s1.pdf]

Global Spatio-temporal Patterns of Influenza in the Post-pandemic Era

Daihai He[1,⋆], Roger Lui[2], Lin Wang[3], Chi Kong Tse[4], Lin Yang[5] and Lewi Stone[6,†]

[1] Department of Applied Mathematics, Hong Kong Polytechnic University, Hong Kong (SAR) China

[2] Department of Mathematical Sciences, Worcester Polytechnic Institute, 100 Institute Road Worcester, MA 01609, United States

[3] School of Public Health, Li Ka Shing Faculty of Medicine, University of Hong Kong, Hong Kong (SAR) China

[4] Department of Electronic and Information Engineering, Hong Kong Polytechnic University Hong Kong (SAR) China

[5] School of Nursing, Hong Kong Polytechnic University, Hong Kong (SAR) China

[6] School of Mathematical and Geospatial Sciences, RMIT University, Melbourne, 3000, Australia

⋆ hedaihai@gmail.com † lewi.stone@rmit.edu.au

Supplementary Materials

S1 Scatter Plot

We showed scatter plots of the skip indexes (for the 2011/12 skip) of the three strains in Fig. S1. Evident negative correlation can be seen between H3N2 and the other two strains.

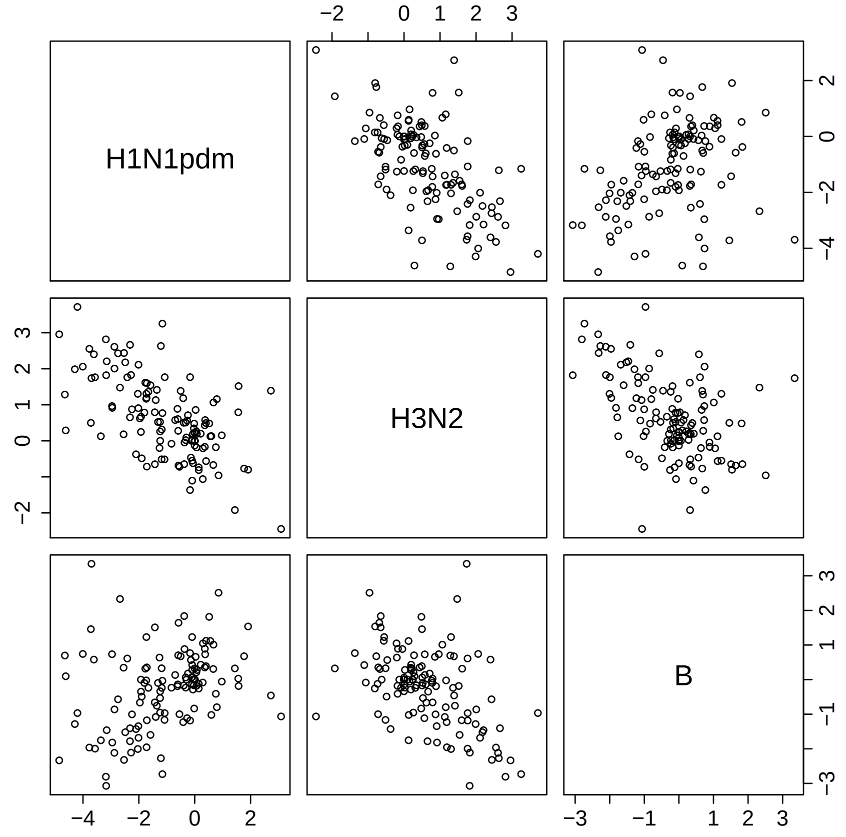

Figure S1: Scatter plot of the skip indexes of the three strains. H3N2 negatively correlates with H1N1pdm and B.

S2 Synchrony Pattern

Figure S2 shows the spatio-temporal patterns of weekly lab-confirmed cases of four types of influenza strains from 35 countries chosen for having the largest total number of cases between January, 2006 and January, 2015. Panels (a) to (d) from top to bottom show the patterns of H3N2, H1N1pdm, H1N1pre, and Flu B, respectively. Countries were listed according to their

latitudes from north to south.

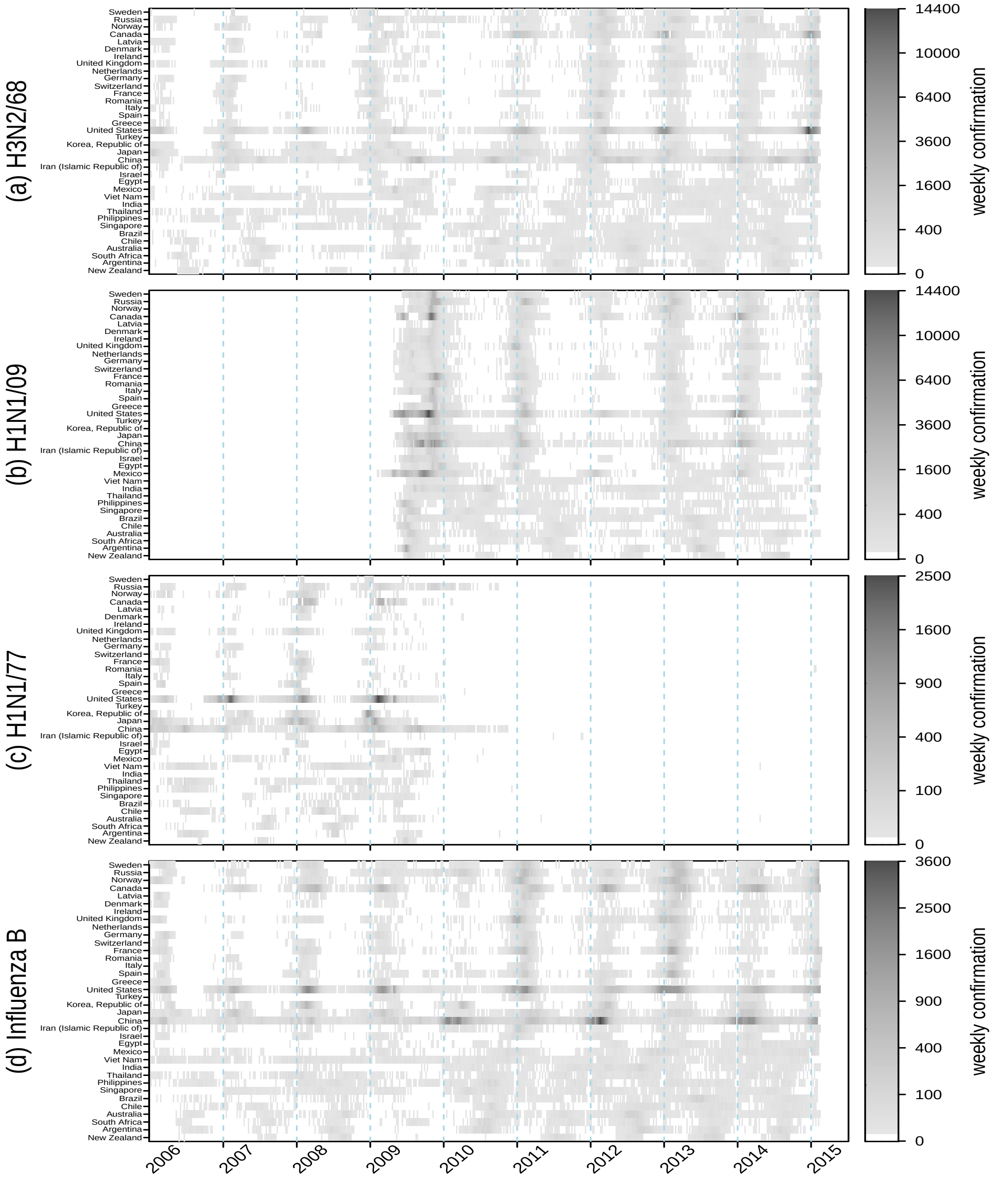

Figure S2: Spatio-temporal patterns of lab-confirmed cases of four types of influenza strains from 35 countries between January, 2006 to January, 2015. Countries are listed from north to south. Grey scale shows the weekly lab-confirmed cases. The horizontal axis is time in weeks. Evident synchrony patterns for H1N1pdm, H3N2 and influenza B can be seen in the temperate countries. H1N1pdm showed skip in most countries in Eastern Asia and Europe during the 2011/12 season. H1N1pre was replaced by H1N1pdm after the 2009 pandemic. Isolated cases of H1N1pre after 2011 are likely due to errors. The color (grey) scheme is in a square-root scale.

Table S1: Synchrony Indicators for countries with at least 50 confirmations for a strain and latitude $\geq$ 29 degree.

Indicator	Season	H1N1	H3N2	Flu B
Median of MCT	2006 - 07	26.6	25.9	28.3
	2007 - 08	23.6	30.6	27.2
	2008 - 09	47.5	24.3	29.7
	2009 - 10	13.0	18.0	30.1
	2010 - 11	23.4	22.4	25.0
	2011 - 12	27.9	27.9	29.6
	2012 - 13	25.8	25.4	28.1
	2013 - 14	27.2	28.0	29.3
SD of MCT	2006 - 07	4.68	3.45	5.00
	2007 - 08	3.92	3.23	2.43
	2008 - 09	7.69	5.81	3.34
	2009 - 10	2.27	10.37	5.29
	2010 - 11	2.18	5.87	3.16
	2011 - 12	4.66	3.76	2.57
	2012 - 13	2.19	2.85	2.89
	2013 - 14	3.06	3.21	3.59
Total of Confirmations	2006 - 07	15706	25430	11048
	2007 - 08	24117	22153	28178
	2008 - 09	136473	35718	22884
	2009 - 10	387627	14565	28296
	2010 - 11	103450	59553	56620
	2011 - 12	9760	84585	45015
	2012 - 13	58850	108576	68944
	2013 - 14	114369	53433	45550
Number of Countries	2006 - 07	8	25	9
	2007 - 08	28	6	23
	2008 - 09	35	28	22
	2009 - 10	47	5	11
	2010 - 11	49	18	47
	2011 - 12	12	47	25
	2012 - 13	49	38	48
	2013 - 14	38	41	19

From Table S1, the standard deviation (SD) of MCT for the H1N1pdm strain in the 2010/11 and 2012/13 seasons are significantly smaller than the other seasons and any of the other strains. In particular, not only the SD of MCT for H1N1pdm in the 2010/11 season is small, but also the total confirmation is high and the number of countries involved is large.

Table S2: Correlations of median/SD of MCT versus the total of confirmations for countries with at least 50 confirmations for a strain and a latitude $\geq$ 29 degree.

Correlation	Total of Confirmations		
	H1N1	H3N2	Flu B
Median of MCT	-0.37	0.21	-0.34
SD of MCT	-0.25	-0.48	-0.53

Thus globally the 2010/11 wave of H1N1pdm was very substantial and well-synchronized. And the countries are more synchronized by H1N1 than by H3N2 or influenza B. This may reflect a more efficient transmissibility of the H1N1pdm virus which allows it to spread more rapidly between countries. These findings corroborate what is observed by eye in Figure 2. Note that the median of MCT of flu B seems larger than the two flu A strains (by two weeks), suggesting that the flu B epidemic lagged behind the other two flu A strains [1].

In Table S2, we showed the correlations between the median (and the sd) of MCT and the total of confirmations for the three strains. Marginally negative correlation is found in most cases except for the median of MCT for H3N2. We calculated these indictors for countries with a latitude $<$ 29 in Tables S3 and S4 and found that the SD of MCT is substantially larger than these northern countries.

Table S3: Synchrony Indicators for countries with more than 50 confirmations for a strain and a latitude < 29 degree.

Indicator	Season	H1N1	H3N2	Flu B
Median of MCT	2006 - 07	40.9	25.7	34.8
	2007 - 08	34.0	33.6	27.0
	2008 - 09	36.2	28.6	26.6
	2009 - 10	24.0	35.6	34.5
	2010 - 11	26.8	36.1	32.4
	2011 - 12	28.4	30.2	30.8
	2012 - 13	28.5	26.8	28.2
	2013 - 14	19.8	28.2	32.2
SD of MCT	2006 - 07	7.55	5.24	8.93
	2007 - 08	8.55	11.34	6.92
	2008 - 09	6.29	8.58	8.53
	2009 - 10	10.18	9.53	8.75
	2010 - 11	10.66	12.30	10.41
	2011 - 12	10.90	9.47	7.17
	2012 - 13	9.06	8.91	8.96
	2013 - 14	7.82	9.01	6.19
Total of Confirmations	2006 - 07	1592	4936	1569
	2007 - 08	3534	2734	4125
	2008 - 09	140065	13251	2063
	2009 - 10	29116	13986	8866
	2010 - 11	16973	11731	8297
	2011 - 12	14379	15961	14086
	2012 - 13	21093	15009	11252
	2013 - 14	16030	17083	10498
Number of Countries	2006 - 07	9	12	10
	2007 - 08	14	10	15
	2008 - 09	39	24	14
	2009 - 10	39	37	31
	2010 - 11	36	44	34
	2011 - 12	34	38	42
	2012 - 13	42	39	39
	2013 - 14	24	44	39

Table S4: Correlations of median/SD of MCT versus the total of confirmations for countries with at least 50 confirmations for a strain and a latitude < 29 degree.

Correlation	Total of Confirmations		
	H1N1	H3N2	Flu B
Median of MCT	0.23	-0.08	0.13
SD of MCT	-0.54	0.09	-0.21

S3 Spatio-temporal Plots of Individual Countries

Figure S3 shows individual plots of 30 populations with the largest number of lab-confirmed cases of H1N1pdm and H3N2 between January, 2009 and January, 2015.

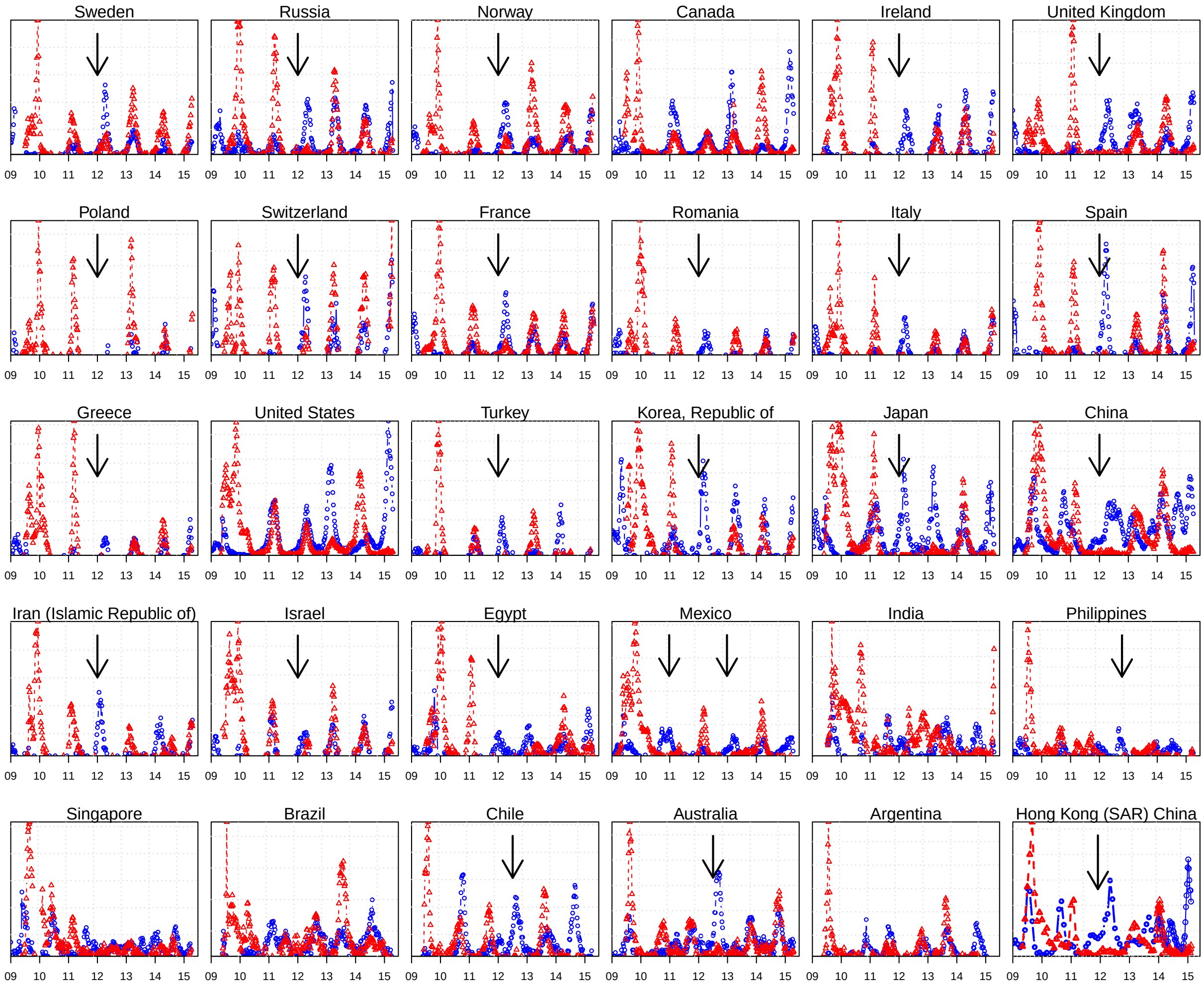

Figure S3: Weekly lab-confirmations of H1N1pdm and H3N2 in 30 populations. Black arrows indicate 'skip' seasons for the H1N1pdm, when the weekly confirmations of H1N1pdm were evidently low or absent.

There are several countries, such as Japan, which skipped both 2011/12 and 2012/13 season. Australia did show sign of skip in the 2012 season. Its skip index is between 1/10 and 1/5. The vaccination coverages in the general population in Australia and Japan were low.

S4 Countries with Regular Annual Patterns

We consider the weekly influenza A lab-confirmations for each country from 1997 to 2013. If there were zeros in the first few years of the time series, these zero time points will be removed. All NA's in the middle are treated as zeros. Then we apply *spectrum* function in R (version 2.15.2) to the time series. We set *span*=(3,5), which are the widths of the modified Daniell smoother. We set other parameters at default values (such as *taper*=0.1). We compare the heights of the spectrum at frequency $f = 1$ and $f < 1$, if the height at $f = 1$ is at 1.75 times of the mean level of $f < 1$, we conclude the time series show regular annual patterns.

S5 Mathematical Model and Likelihood Functions

We consider a compartmental (Susceptible-Exposed-Infectious-Recovered) model in the main text,

$$\dot{S} = \lambda R - \beta(t) S I - v(t) S \quad (1a)$$

$$\dot{E} = \beta(t) S I - \sigma E \quad (1b)$$

$$\dot{I} = \sigma E - \gamma I \quad (1c)$$

$$\dot{R} = \gamma I - \lambda R + v(t) S \quad (1d)$$

where S, E, I, and R denote the proportions of susceptible, exposed, infectious and recovered proportions in a population, and $S + E + I + R = 1$; In particular, Exposed refers to those infected but not yet infectious; λ, $\beta(t)$ and $v(t)$ denote rates of individuals moving from Recovered to Susceptible (due to loss-of-immunity), the transmission rates of the virus, and the vaccination rate (individuals moving from Susceptible to Recovered, after taking account into the effectiveness of the vaccine and the delay between the vaccination and being effective of the vaccine). σ (and γ) denotes the rates of individuals moving from Exposed (and Infectious)

to Infectious (and Recovered).

It would be very challenging to disentangle $\beta(t)$, λ and $v(t)$, given that we assume $\beta(t)$ is time-varying. Mathematically, $v(t)$ can be transferred into as a variation in the $\beta(t)$ [2]. But we can roughly estimate the $v(t)$ from other sources (e.g. number of vaccines delivered and the time of the vaccination etc). For simplicity in this manuscript, we set $v(t) = 0$, and leave this challenging task to future works.

The weekly report cases is a weekly integral in the form

$$Z_t = \int_{\text{a week}} \rho(t)\gamma I dt \tag{2}$$

We assume that the observed weekly lab-confirmations C_t is a random sample from a Negative-binomial (NB) distribution

$$C_t \sim \text{NB}\left(n = \frac{1}{\tau}, p = \frac{1}{1 + Z_t\tau}\right) \tag{3}$$

where n and p denote the size and probability of the NB distribution (R version 2.15.2), and τ denotes an over-dispersion parameter which will be estimated. (In the sense that the NB can be viewed as an over-dispersed Poisson process.) The mean and variance of the NB distribution,

$$mean = \frac{n(1-p)}{p} = Z_t \tag{4a}$$

$$variance = \frac{n(1-p)}{p^2} = Z_t(1 + Z_t\tau) \tag{4b}$$

When $\tau = 0$, the NB distribution is reduced to a Poisson distribution. Thus the likelihood for the week (l_t) can be simply calculated, namely the probability of observing C_t, given Z_t and τ, under the NB distribution [3]. The overall likelihood function is

$$L(\theta|C_{0,\ldots,N}) = \prod_{t=0}^{N} l_t \tag{5}$$

where θ denotes the parameter vector. We used iterated filtering to estimate the maximum likelihood estimates for θ. This methodology has been extensively studied and used in a number of publications [4, 5, 6, 7, 8, 9, 10]

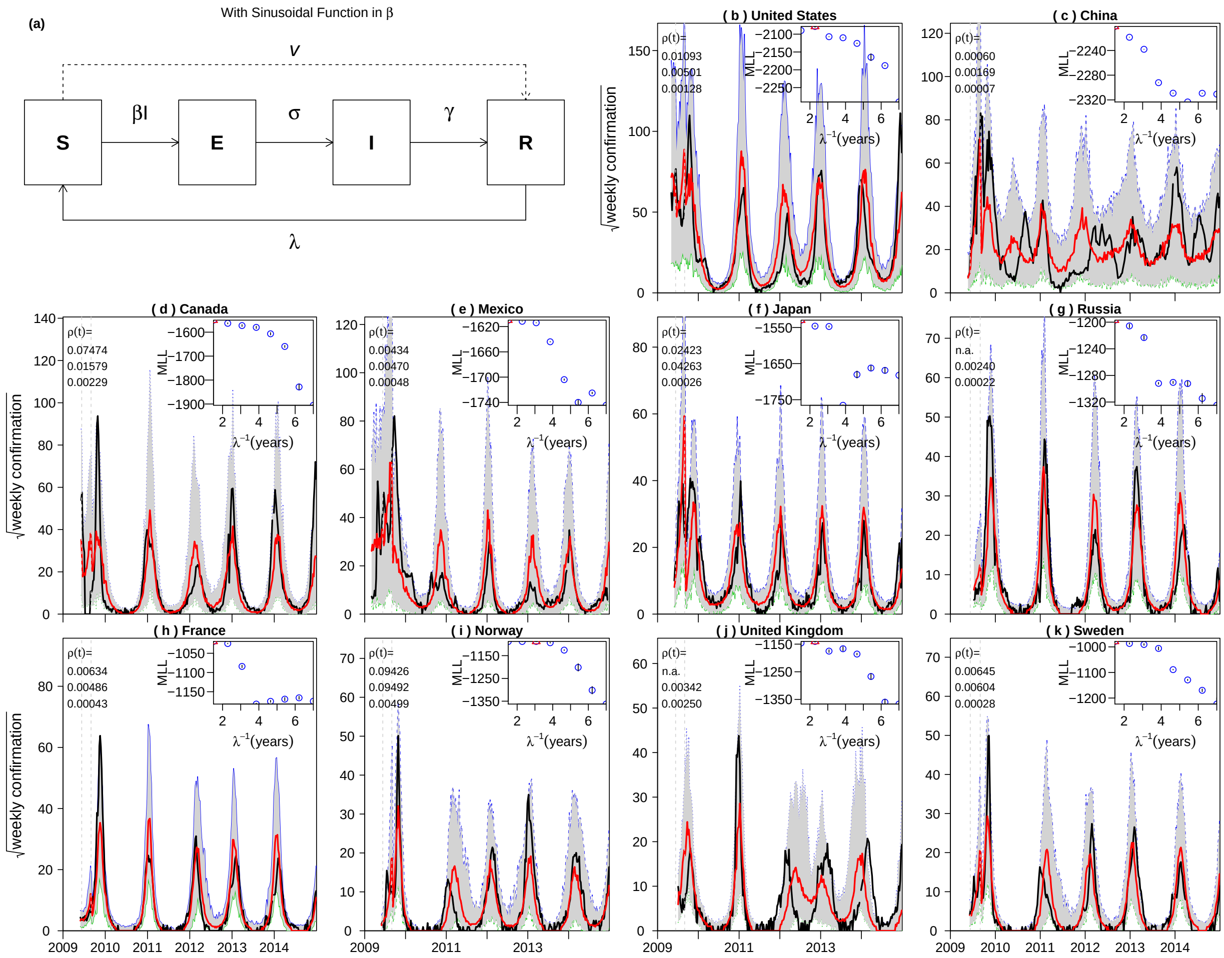

Figure S4: Fitting an SEIR model, with a sinusoidal function in the transmission rate, to influenza A confirmations in 10 countries. Panel **a**, a flowchart of the model. Panel (**b-k**), the results in 10 countries. Each panel shows the simulation (red) versus the observed (black), with the best fitting parameters. The dotted vertical lines indicate the two timings for the reporting ratio changes. The simulations are median values for each week of 1000 simulations and shaded region show the 95% range. The inset panel shows the profile log-likelihood for the duration of immunity.

In Table S5, we show the AIC_c difference between models with a sinusoidal function and a cubic-spline function($n = 6$). The sinusoidal model has three less parameters than the cubic-spline model ($n = 6$). The AIC_c values of the sinusoidal model are substantially larger than the cubic-spline model expect for one country – Norway. The cubic-spline ($n = 6$) model

performs better than the sinusoidal model in nine out of ten countries.

The small-sample-size corrected Akaike Information Criterion ($AICc$) is used to measure goodness-of-fit for models:

$$AICc = -2\log L + \frac{2kN}{N-k-1} \tag{6}$$

where N denotes number of data points and k denotes number of free parameters. As the $AICc$ should take into account the number of model parameters (through penalization), this should indicate that the improved fit of the transmission function is not due to the extra parameters associated with over-fitting problems.

Table S5: A Model comparison and parameter estimates. The ΔAIC_c denotes the difference between models with a sinusoidal function and a cubic-spline function ($n = 6$). $\mathcal{R}_0$ and $S(0)$ are estimated with the cubic-spline model.

		Cubic-spline		Sinusoid	
Country	ΔAIC_c	$\mathcal{R}_0$	$S(0)$	$\mathcal{R}_0$	$S(0)$
United States	71	1.68	0.63	1.92	0.55
China	245.4	2.9	0.41	2.55	0.42
Canada	89.2	1.89	0.58	1.73	0.62
Mexico	17.2	1.83	0.55	1.8	0.58
Japan	88.6	1.84	0.62	1.99	0.57
Russia	20	1.79	0.6	1.94	0.6
France	5.9	2.01	0.53	2.03	0.55
Norway	-9	1.82	0.7	2.65	0.47
United Kingdom	89.9	2.82	0.4	2.15	0.56
Sweden	55.2	2	0.58	1.88	0.64

S6 Influenza Vaccination Coverage

Table S6: Estimated influenza vaccination coverage in the general population

Country/ Population group	2008-09	2009-10	H1N1pdm (2009-10)	2010-11	2011-12
Austria			3		
Bulgaria	4.74	6.33		3.41	2.91
Cyprus	11.8	16.1	3	11.9	12.05
Czech Republic	7.2	7.8	0.6		
Denmark			NA		
England			NA		
Estonia			3	1.3	
Finland			50		
France		20.6	8		
Germany	28.1	26.6	8		
Greece			3		
Hungary			27	10.2	10.3
Iceland	16.2	17.7	46	14.9	14.2
Ireland			23		
Italy	19.1	19.6	4	17.2	17.8
Latvia	0.9	0.8		0.5	0.4
Lithuania	7.8	4		3	6.4
Luxembourg	16.6	18.1	6		
Malta			23		
The Netherlands	21.9	22.4	30	21.3	65.7
Norway	12	12(13)	45	12	9
Poland	4.1	3.1		3.1	4.5
Portugal	15	15(19)	6	17.5	16.4
Romania	-	5.2	9	5.6	3
Slovakia	12.8	12.4	0.4	9.1	7.5
Slovenia	7.3	7.3	5		4.89
Spain			27		
Sweden			59		

Source:
`http://venice.cineca.org/Final_Seasonal_Influenza_Vaccination_Survey_2010.pdf`
`http://www.eurosurveillance.org/ViewArticle.aspx?ArticleId=20064`
`http://ecdc.europa.eu/en/escaide/materials/presentations%202010/escaide2010_late_breakers_mereckiene.pdf`

Table S7 shows the comparison of influenza virus vaccination coverage in the United States and Canada.

Table S7: Influenza vaccination coverage in U.S. ($>$ 6 months) and Canada ($>$ 12 years old)

Country	2009-2010 (seasonal)	H1N1pdm	2009-2010 (combined)	2010-11	2011-12
US	41.2	27.2	47.8	43.0	41.8 (47.6?)
Canada	32.2	41.3	NA	30.2	28.9

Source:
http://www.cdc.gov/flu/professionals/vaccination/coverage_0910estimates.htm.
http://www.statcan.gc.ca/pub/82-003-x/2010004/article/11348/tbl/tbl01-eng.htm,
http://www.statcan.gc.ca/tables-tableaux/sum-som/l01/cst01/health101b-eng.htm

In Japan, a study in four wards (communities) and one city in Tokyo showed that, among those between age 18 and 65, 38.1% received seasonal influenza vaccine and 12.1% received A(H1N1pdm) influenza vaccine between October, 2009 and April, 2010 [11]. The vaccination coverage for H1N1pdm in Hong Kong is around 14% http://www.dh.gov.hk/.

S7 Spatio-temporal Pattern from 1995 to 2005

Figure S5 shows the spatio-temporal pattern of three strains from 1995 to 2005, and that of the weekly total specimens processed.

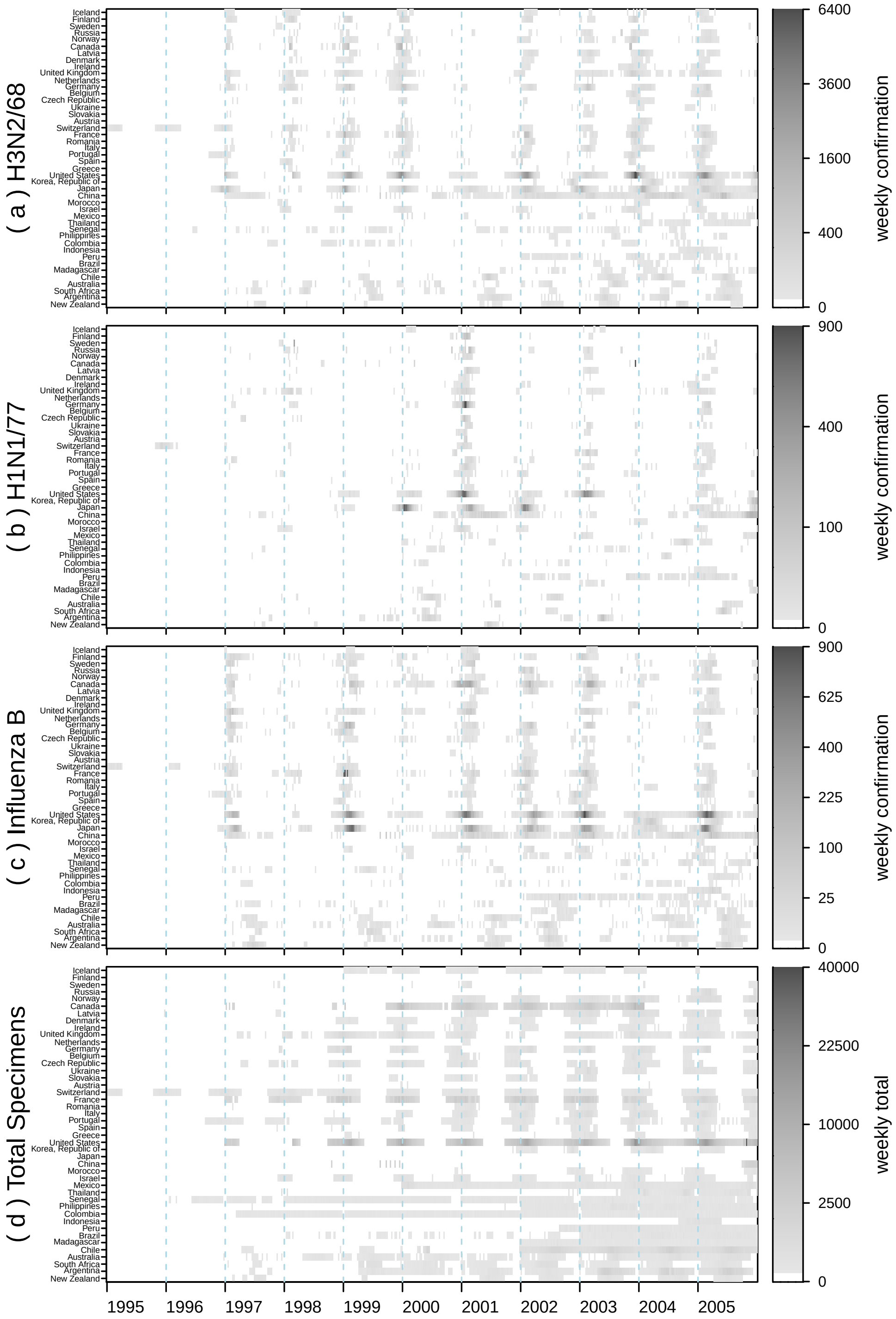

Figure S5: Spatio-temporal patterns of lab-confirmed cases of three types of influenza strains and total specimens processed for 44 countries which reported the largest confirmations between January, 1995 and December, 2005. Countries are listed in order of their latitudes. Grey scale shows the weekly lab-confirmed cases after taking square root. The horizontal axis is time in weeks.

S8 Vaccine Components

Influenza virus vaccine composition is an indicator of predicted dominance of influenza strains. Table S8 shows the influenza virus vaccine composition recommended by the Food and Drug Administration (FDA) of the United States (http://www.fda.gov/BiologicsBloodVaccines/GuidanceComplianceRegulatoryInformation/Post-MarketActivities/LotReleases/ucm062928.htm), which is usually the same as the recommendation by the World Health Organization for the Northern Hemisphere countries (http://www.who.int/influenza/vaccines/virus/recommendations/en/).

Table S8 is modified from http://www.hpa.org.uk/Topics/InfectiousDiseases/InfectionsAZ/SeasonalInfluenza/.

Table S8: Influenza vaccine composition recommended by U.S. Food and Drug Administration

	Vaccine Strains			UK Circulating Strains		
Season	A(H3N2)	A(H1N1)	B	A(H3N2)	A(H1N1)	B
1968/69	A/Hong Kong/1/68			A/Hong Kong/1/68		
1969/70	A/Hong Kong/1/68			A/Hong Kong/1/68		
1970/71	A/Hong Kong/1/68			A/Hong Kong/1/68		
1971/72	A/Hong Kong/1/68			A/Hong Kong/1/68		
	A/England/42/72			A/England/42/72		B/Hong Kong/5/72
1973/74	A/England/42/72		B/Victoria/98926/70	A/Port Chalmers/1/73		B/Hong Kong/5/72
			B/Hong Kong/5/72			
1974/75	A/Port Chalmers/1/73		B/Hong Kong/5/72	A/Port Chalmers/1/73		
1975/76	A/Port Chalmers/1/73	A/Scotland/840/74	B/Hong Kong/5/72	A/Victoria/3/75		B/Hong Kong/5/72
1976/77	A/Victoria/3/75		B/Hong Kong/5/72	A/Victoria/3/75		B/Hong Kong/8/73
1977/78	A/Victoria/3/75		B/Hong Kong/5/72	A/Texas/1/77	A/USSR/90/77	
1978/79	A/Texas/1/77	A/USSR/90/77	B/Hong Kong/5/72	A/Texas/1/77		B/Hong Kong/8/73
1979/80	A/Texas/1/77	A/USSR/90/77	B/Hong Kong/5/72	A/Bangkok/1/79		B/Singapore/222/79
						B/Singapore/263/79
1980/81	A/Bangkok/1/79	A/USSR/90/77	B/Singapore/222/79	A/Bangkok/1/79		B/Singapore/222/79
		A/Brazil/11/78				
1981/82	A/Bangkok/1/79	A/Brazil/11/78	B/Singapore/222/79	A/Bangkok/1/79		
1982/83	A/Bangkok/1/79	A/Brazil/11/78	B/Singapore/222/79	A/Philippines/2/82	A/Brazil/11/78	B/Singapore/222/79
1983/84	A/Philippines/2/82	A/Brazil/11/78	B/Singapore/222/79	A/Philippines/2/82		
1984/85	A/Philippines/2/82	A/Chile/1/83	B/USSR/100/83	A/Philippines/2/82		
1985/86	A/Philippines/2/82	A/Chile/1/83	B/USSR/100/83	A/Mississippi/1/85		
1986/87	A/Christchurch/4/85	A/Chile/1/83	B/Ann Arbor/1/86	A/Christchurch/4/85-		
	A/Mississippi/1/85	A/Singapore/6/86				
1987/88	A/Leningrad/360/86	A/Singapore/6/86	B/Ann Arbor/1/86	A/Sichuan/2/87	A/Singapore/6/86	B/Beijing/1/87
1988/89	A/Sichuan/2/87	A/Singapore/6/86	B/Beijing/1/87	A/Shanghai/11/87	A/Singapore/6/86	B/Beijing/1/87
1989/90	A/Shanghai/11/87	A/Singapore/6/86	B/Yamagata/16/88	A/Shanghai/11/87	A/Singapore/6/86	
			or B/Panama/45/90			B/Yamagata/16/88
1990/91	A/Guizhou/54/89	A/Singapore/6/86	B/Yamagata/16/88	A/Beijing/353/89	A/Singapore/6/86	B/Yamagata/16/88
			or B/Panama/45/90		A/Victoria/36/88	
1991/92	A/Beijing/353/89	A/Singapore/6/86	B/Yamagata/16/880	A/Beijing/353/89	A/Singapore/6/86	B/Yamagata/16/88
			or B/Panama/45/90		A/Victoria/36/88	B/Panama/45/90
1992/93	A/Beijing/353/89	A/Singapore/6/86	B/Panama/45/90	A/Beijing/32/92	A/Singapore/6/86	B/Panama/45/90
					A/Victoria/36/88	B/Quingdao/102/91
1993/94	A/Beijing/32/92	A/Singapore/6/86	B/Panama/45/90	A/Shangdong/9/93		B/Panama/45/90
1994/95	A/Shangdong/9/93	A/Singapore/6/86	B/Panama/45/90	A/Johannesburg/33/94	A/Singapore/6/86	B/Beijing/184/93
					A/Texas/36/91	
1995/96	A/Johannesburg/33/94	A/Singapore/6/86	B/Beijing/184/93	A/Johannesburg/33/94	A/Singapore/6/86	B/Beijing/184/93
					A/Texas/36/91	
1996/97	A/Wuhan/359/95	A/Singapore/6/86	B/Beijing/184/93	A/Wuhan/359/95	A/Bayern/7/95	B/Beijing/184/93
1997/98	A/Wuhan/359/95	A/Bayern/7/95	B/Beijing/184/93	A/Sydney/5/97	A/Bayern/7/95	B/Beijing/184/93
1998/99	A/Sydney/5/97	A/Beijing/262/95	B/Beijing/184/93	A/Sydney/5/97	A/Bayern/7/95	B/Beijing/184/93
1999/00	A/Sydney/5/97	A/Beijing/262/95	B/Beijing/184/93	A/Sydney/5/97	A/Bayern/7/95	B/Beijing/184/93
2000/01	A/Moscow/10/99	A/New Caledonia/20/99	B/Beijing/184/93		A/New Caledonia/20/99	B/Sichuan/379/99
2001/02	A/Moscow/10/99	A/New Caledonia/20/99	B/Sichuan/379/99	A/Moscow/10/99	A/New Caledonia/20/99	B/Sichuan/379/99
					A/NewCaledonia/10/99 (H1N2)	
2002/03	A/Moscow/10/99	A/New Caledonia/20/99	B/HongKong/330/2001	A/Moscow/10/99	A/New Caledonia/20/99	B/HongKong/330/2001
2003/04	A/Moscow/10/99	A/New Caledonia/20/99	B/HongKong/330/2001	A/Fujian/411/2002	A/New Caledonia/20/99	
2004/05	A/Fujian/411/2002	A/New Caledonia/20/99	B/Shanghai/361/2002	A/Wellington/1/2004	A/New Caledonia/20/99	B/Shanghai/361/2002
2005/06	A/California/7/2004	A/New Caledonia/20/99	B/Shanghai/361/2002	A/California/7/2004	A/New Caledonia/20/99	B/HongKong/330/2001
2006/07	A/Wisconsin/67/2005	A/New Caledonia/20/99	B/Malaysia/2506/2004	A/Wisconsin/67/2005	A/New Caledonia/20/99	
2007/08	A/Wisconsin/67/2005	A/Solomon Islands/3/2006	B/Malaysia/2506/2004	A/Wisconsin/67/2005	A/Solomon Islands/3/2006	B/Florida/4/2006
2008/09	A/Brisbane/10/2007	A/Brisbane/59/2007	B/Florida/4/2006	A/Brisbane/10/2007	A/Brisbane/59/2007	B/Florida/4/2006
					A/California/07/2009 from Apr-2009	B/Malaysia/2506/2004
2009/10	A/Brisbane/10/2007	A/Brisbane/59/2007	B/Brisbane/60/2008	A/Perth/16/2009	A/California/07/2009	B/Brisbane/60/2008
2010/11	A/Perth/16/2009	A/California/07/2009	B/Brisbane/60/2008	A/Perth/16/2009	A/California/07/2009	B/Brisbane/60/2008
2011/12	A/Perth/16/2009	A/California/7/2009	B/Brisbane/60/2008	A/Perth/16/2009	A/California/7/2009	B-Victoria/B-Yamagata
2012/13	A/Victoria /361/2011	A/California/7/2009	B/Wisconsin /1/2010	A/Victoria/361/2011	A/California/7/2009	B/Wisconsin/1/2010
2013/14	A/Texas/50/2012	A/California/7/2009	B/Massachusetts/2/2012	A/Texas/50/2012	A/California/7/2009	B-Yamagata

S9 Comparisons among the US, UK and Canada

Figure S6 shows the comparisons of the population standardized weekly confirmations (confirmations per 100,000 inhabitants) among the United States, United Kingdom and Canada. These comparisons suggest that the attack rate of H1N1pdm in 2009 might be low in UK than in North America. The 2011/12 skip of H1N1pdm is evident in United Kingdom. In 2014/15 flu season, H1N1pdm skipped in all of the three countries.

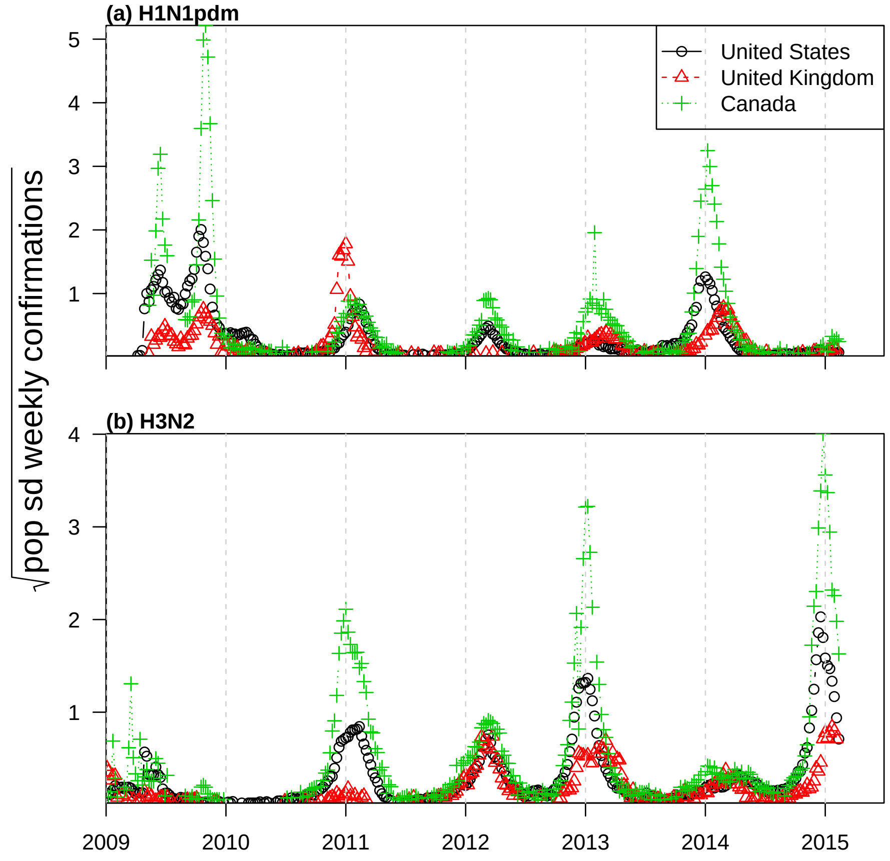

Figure S6: Comparison of the population standardized weekly confirmations between US, UK and Canada for H1N1pdm and H3N2.

S10 A list of countries

Regions and sub-regions are from http://millenniumindicators.un.org/unsd/methods/m49/m49regin.htm.

Table S9:

name	subregion	pop2005	latitude
Mauritius	Eastern Africa	1241173	-20.255
Madagascar	Eastern Africa	18642586	-19.374
Zambia	Eastern Africa	11478317	-14.614
Mozambique	Eastern Africa	20532675	-14.422
United Republic of Tanzania	Eastern Africa	38477873	-6.270
Rwanda	Eastern Africa	9233793	-1.998
Kenya	Eastern Africa	35598952	0.530
Uganda	Eastern Africa	28947181	1.280
Ethiopia	Eastern Africa	78985857	8.626
Angola	Middle Africa	16095214	-12.296
Democratic Republic of the Congo	Middle Africa	58740547	-2.876
Congo	Middle Africa	3609851	-0.055
Cameroon	Middle Africa	17795149	5.133
Central African Republic	Middle Africa	4191429	6.571
Chad	Middle Africa	10145609	15.361
Sudan	Northern Africa	36899747	13.832
Egypt	Northern Africa	72849793	26.494
Algeria	Northern Africa	32854159	28.163
Morocco	Northern Africa	30494991	32.706
Tunisia	Northern Africa	10104685	35.383
South Africa	Southern Africa	47938663	-30.558
Cote d'Ivoire	Western Africa	18584701	7.632
Ghana	Western Africa	2253501	7.960
Sierra Leone	Western Africa	5586403	8.560
Togo	Western Africa	6238572	8.799
Nigeria	Western Africa	141356083	9.594
Guinea	Western Africa	9002656	10.439
Guinea-Bissau	Western Africa	1596929	12.125
Burkina Faso	Western Africa	13933363	12.278
Senegal	Western Africa	1177034	15.013
Cape Verde	Western Africa	506807	15.071
Mali	Western Africa	1161109	17.350

Table S10:

name	subregion	pop2005	latitude
Niger	Western Africa	1326419	17.426
Mauritania	Western Africa	2963105	20.260
United States	Northern America	299846449	39.622
Canada	Northern America	32270507	59.081
Saint Lucia	Caribbean	16124	13.898
Martinique	Caribbean	395896	14.653
Guadeloupe	Caribbean	438403	16.286
Saint Martin	Caribbean	0	18.094
Jamaica	Caribbean	2682469	18.151
Dominican Republic	Caribbean	9469601	19.015
Cuba	Caribbean	11259905	21.297
Panama	Central America	3231502	8.384
Costa Rica	Central America	4327228	9.971
Nicaragua	Central America	5462539	12.840
El Salvador	Central America	6668356	13.736
Honduras	Central America	683411	14.819
Guatemala	Central America	12709564	15.256
Mexico	Central America	104266392	23.951
Argentina	South America	38747148	-35.377
Uruguay	South America	3325727	-32.800
Chile	South America	16295102	-23.389
Paraguay	South America	5904342	-23.236
Bolivia	South America	9182015	-16.715
Brazil	South America	186830759	-10.772
Peru	South America	27274266	-9.326
Ecuador	South America	13060993	-1.385
Colombia	South America	4494579	3.900
French Guiana	South America	192099	3.924
Suriname	South America	452468	4.127
Venezuela	South America	26725573	7.125
Kyrgyzstan	Central Asia	5203547	41.465
Uzbekistan	Central Asia	26593123	41.750

Table S11:

name	subregion	pop2005	latitude
Kazakhstan	Central Asia	15210609	48.160
China	Eastern Asia	1312978855	33.420
Japan	Eastern Asia	127896740	36.491
Korea, Republic of	Eastern Asia	47869837	36.504
Mongolia	Eastern Asia	2580704	46.056
Sri Lanka	Southern Asia	19120763	7.612
India	Southern Asia	1134403141	21.000
Bangladesh	Southern Asia	15328112	24.218
Bhutan	Southern Asia	637013	27.415
Nepal	Southern Asia	27093656	28.253
Pakistan	Southern Asia	158080591	29.967
Iran (Islamic Republic of)	Southern Asia	69420607	32.565
Afghanistan	Southern Asia	25067407	33.677
Indonesia	South-Eastern Asia	226063044	-0.976
Singapore	South-Eastern Asia	4327468	1.351
Malaysia	South-Eastern Asia	25652985	4.201
Philippines	South-Eastern Asia	84566163	11.118
Cambodia	South-Eastern Asia	13955507	12.714
Thailand	South-Eastern Asia	63002911	15.700
Lao People's Democratic Republic	South-Eastern Asia	566391	19.905
Viet Nam	South-Eastern Asia	85028643	21.491
Oman	Western Asia	2507042	21.656
Qatar	Western Asia	796186	25.316
Bahrain	Western Asia	724788	26.019
Jordan	Western Asia	5544066	30.703
Israel	Western Asia	6692037	31.026
Iraq	Western Asia	27995984	33.048
Syrian Arab Republic	Western Asia	18893881	35.013
Turkey	Western Asia	72969723	39.061
Azerbaijan	Western Asia	8352021	40.430
Armenia	Western Asia	3017661	40.534
Georgia	Western Asia	4473409	42.176

Table S12:

name	subregion	pop2005	latitude
Bulgaria	Eastern Europe	7744591	42.761
Romania	Eastern Europe	21627557	45.844
Hungary	Eastern Europe	10086387	47.070
Republic of Moldova	Eastern Europe	3876661	47.193
Slovakia	Eastern Europe	5386995	48.707
Ukraine	Eastern Europe	46917544	49.016
Czech Republic	Eastern Europe	10191762	49.743
Poland	Eastern Europe	38195558	52.125
Belarus	Eastern Europe	9795287	53.540
Russia	Eastern Europe	143953092	61.988
United Kingdom	Northern Europe	60244834	53.000
Ireland	Northern Europe	4143294	53.177
Lithuania	Northern Europe	3425077	55.336
Denmark	Northern Europe	5416945	56.058
Latvia	Northern Europe	2301793	56.858
Estonia	Northern Europe	1344312	58.674
Norway	Northern Europe	4638836	61.152
Sweden	Northern Europe	9038049	62.011
Finland	Northern Europe	5246004	64.504
Iceland	Northern Europe	295732	64.764
Malta	Southern Europe	402617	35.890
Greece	Southern Europe	11099737	39.666
Spain	Southern Europe	43397491	40.227
Portugal	Southern Europe	10528226	40.309
Albania	Southern Europe	3153731	41.143
The former Yugoslav Republic of Macedonia	Southern Europe	2033655	41.600
Italy	Southern Europe	5864636	42.700
Serbia	Southern Europe	9863026	44.032
Bosnia and Herzegovina	Southern Europe	3915238	44.169
Croatia	Southern Europe	455149	45.723
Slovenia	Southern Europe	1999425	46.124
France	Western Europe	60990544	46.565

Table S13:

name	subregion	pop2005	latitude
Switzerland	Western Europe	7424389	46.861
Austria	Western Europe	8291979	47.683
Luxembourg	Western Europe	456613	49.771
Belgium	Western Europe	10398049	50.643
Germany	Western Europe	82652369	51.110
Netherlands	Western Europe	1632769	52.077
New Zealand	Australia and New Zealand	4097112	-42.634
Australia	Australia and New Zealand	20310208	-24.973
New Caledonia	Melanesia	234185	-21.359
Fiji	Melanesia	828046	-17.819

References

[1] Finkelman, B. S. *et al.* Global patterns in seasonal activity of influenza A/H3N2, A/H1N1, and B from 1997 to 2005: Viral coexistence and latitudinal gradients. *PLoS ONE* **2**, e1296 (2007). URL `http://www.plosone.org/article/info%3Adoi%2F10.1371%2Fjournal.pone.0001296`.

[2] Earn, D. J., Rohani, P., Bolker, B. M. & Grenfell, B. T. A simple model for complex dynamical transitions in epidemics. *Science* **287**, 667–670 (2000).

[3] Breto, C., He, D. H., Ionides, E. L. & King, A. A. Time series analysis via mechanistic models. *Ann Appl Stat* **3**, 319–348 (2009).

[4] Ionides, E., Bretó, C. & King, A. Inference for nonlinear dynamical systems. *Proceedings of the National Academy of Sciences* **103**, 18438–18443 (2006).

[5] Ionides, E. L., Bhadra, A., Atchadé, Y., King, A. *et al.* Iterated filtering. *The Annals of Statistics* **39**, 1776–1802 (2011).

[6] Earn, D. J. D. *et al.* Effects of school closure on incidence of pandemic influenza in alberta, canada. *Ann Intern Med* **156**, 173–U32 (2012).

[7] Camacho, A. *et al.* Explaining rapid reinfections in multiple-wave influenza outbreaks: Tristan da cunha 1971 epidemic as a case study. *Proceedings of the Royal Society B: Biological Sciences* **278**, 3635–3643 (2011).

[8] He, D. H., Dushoff, J., Day, T., Ma, J. L. & Earn, D. J. D. Mechanistic modelling of the three waves of the 1918 influenza pandemic. *Theor Ecol* **4**, 283–288 (2011).

[9] He, D. H., Ionides, E. L. & King, A. A. Plug-and-play inference for disease dynamics: measles in large and small populations as a case study. *J R Soc Interface* **7**, 271–283 (2010).

[10] King, A. A., Ionides, E. L., Pascual, M. & Bouma, M. J. Inapparent infections and cholera dynamics. *Nature* **454**, 877–880 (2008).

[11] Yi, S., Nonaka, D., Nomoto, M., Kobayashi, J. & Mizoue, T. Predictors of the Uptake of A (H1N1) Influenza Vaccine: Findings from a Population-Based Longitudinal Study in Tokyo. *PLoS ONE* **6**, e18893 (2011). URL http://www.plosone.org/article/info%3Adoi%2F10.1371%2Fjournal.pone.0018893.
